# Study of Polysulfone-Impregnated Hydroxyapatite for Ultrafiltration in Whey Protein Separation

**DOI:** 10.3390/polym16213079

**Published:** 2024-10-31

**Authors:** Tutik Sriani, Muslim Mahardika, Budi Arifvianto, Farazila Yusof, Yudan Whulanza, Gunawan Setia Prihandana, Ario Sunar Baskoro

**Affiliations:** 1 Department of Research and Development, PT. Global Meditek Utama-IITOYA, Sardonoharjo, Ngaglik, Sleman, Yogyakarta 55581, Indonesia; tsriani@gmail.com; 2 Department of Mechanical and Industrial Engineering, Faculty of Engineering, Universitas Gadjah Mada, Jalan Grafika No. 2, Yogyakarta 55281, Indonesia; muslim_mahardika@ugm.ac.id (M.M.); budi.arif@ugm.ac.id (B.A.); 3 Centre of Advanced Manufacturing & Material Processing (AMMP Centre), Department of Mechanical Engineering, Faculty of Engineering, Universiti Malaya, Kuala Lumpur 50603, Malaysia; farazila@um.edu.my; 4 Centre for Foundation Studies in Science, University Malaya, Kuala Lumpur 50603, Malaysia; 5 Department of Mechanical Engineering, Faculty of Engineering, Universitas Indonesia, Kampus UI, Depok 16425, Indonesia; yudan@eng.ui.ac.id; 6 Department of Industrial Engineering, Faculty of Advanced Technology and Multidiscipline, Universitas Airlangga, Jl. Dr. Ir. H. Soekarno, Surabaya 60115, Indonesia

**Keywords:** polysulfone, hydroxyapatite, ultrafiltration, whey, clean water, protein separation

## Abstract

Polysulfone (Psf) ultrafiltration flat-sheet membranes were modified with hydroxyapatite (HA) powder during preparation using the wet-phase inversion method. HA was incorporated to enhance the protein separation capabilities. The asymmetric Psf membranes were synthesized using NMP as the solvent. Through Scanning Electron Microscopy (SEM) analysis, it was revealed that HA was distributed across the membrane. Incorporating HA led to higher flux, the improved rejection of protein, and enhanced surface hydrophilicity. The permeability flux increased with HA concentration, peaking at 0.3 wt.%, resulting in a 38% improvement to 65 LMH/bar. Whey protein separation was evaluated using the model proteins BSA and lysozyme, representing α-Lactalbumin. The results of protein rejection for the blend membranes indicated that the rejection rates for BSA and lysozyme increased to 97.2% and 73%, respectively. Both the native and blend membranes showed similar BSA rejection rates; however, the blend membranes demonstrated better performance in lysozyme separation, indicating superior selectivity compared to native membranes. The modified membranes exhibited improved hydrophilicity, with water contact angles decreasing from 66° to 53°, alongside improved antifouling properties, indicated by a lower flux decline ratio value. This simple and economical modification method enhances permeability without sacrificing separation efficiency, hence facilitating the scalability of membrane production in the whey protein separation industry.

## 1. Introduction

Regular bovine milk comprises at least 3.5% fat, 32 g/L of proteins and an approximately 78% fraction of caseins, while the remaining consists of whey proteins [1]. The dairy business produces a substantial amount of whey, predominantly seen as waste, primarily originating from cheese production [2]. Isolating and refining whey proteins enhances the value of what would otherwise be considered waste, hence increasing the sustainability and profitability of the dairy sector [3]. Whey is regarded as a complete protein, encompassing all essential amino acids. Whey protein consists of multiple distinct proteins, each with unique characteristics and advantages. β-Lactoglobulin constitutes the predominant whey protein, comprising approximately 50–55% of the total protein content, essential for muscle recovery and growth owing to its high concentration of branched-chain amino acids [4]. α-Lactalbumin comprises around 20–25% of whey protein, consists of 123 amino acids, and contains tryptophan, which aids in serotonin generation and may enhance relaxation [5]. Glycomacropeptide (GMP) constitutes around 10–15% of whey protein, with 64 amino acids, which function to trigger hormones that enhance satiety and facilitate digestion [6]. Immunoglobulins, bovine serum albumin (BSA), and lactoferrin comprise around 10%, 5% to 10%, and less than 1% of whey protein, respectively [7]. Whey also comprises several proteins found at relatively low concentrations that exhibit significant biological action [8]. Protein powders are considered a quick and high-quality protein source for enhancing post-exercise recovery [9]. Given that each protein type offers distinct advantages, it is essential to isolate each protein to optimize its benefits.

Membrane systems are extensively used in the dairy industry for efficiently regulating the lactose, fat, and protein content of various products due to their highly efficient separation and purification methods [10]. Ultrafiltration (UF) employs polymeric or ceramic membranes that completely retain whey proteins, effectively eliminating superfluous water and lactose [11]. Polysulfone (Psf) is distinguished as a preferred polymer for ultrafiltration membrane fabrication owing to its remarkable physicochemical characteristics [12]. Psf membranes exhibit resistance to numerous contaminants, endure a wide temperature range while preserving their integrity, possess favorable electrical properties, and are easy to fabricate [13]. Nonetheless, despite their beneficial characteristics, Psf membranes are susceptible to fouling, which leads to a reduction in water flux as it advances [14]. The primary issue encountered in the separation of whey proteins is membrane fouling. Researchers frequently incorporate chemicals during membrane manufacture to produce membranes with enhanced characteristics, such as exceptional water permeability, effective rejection, and anti-fouling surfaces. Various additives/fillers have been examined to enhance the anti-fouling characteristics of UF membranes, including polydopamine nanofillers [15], metal oxide nanoparticles [16], carbon-dot-grafted silica [17], graphene oxide [18], hydroxyapatite nanotubes [19], and others.

Hydroxyapatite (HA) has garnered significant attention in contemporary material chemistry owing to its exceptional biocompatibility, eco-friendliness, cost-effectiveness, and abundance of hydroxyl groups. Ouda et al. [15] incorporated hyaluronic acid (HA) and polydopamine (PDA) into polylactic acid membranes, discovering that the enhancement in flux and membrane hydrophilicity resulted from the addition of HA powder and the application of a PDA coating. Instead of using PDA, Anwar and Arthanareeswaran [20] applied silver as an addition to the polyphenylsulfone membrane, coating hydroxyapatite (HA) to treat Palm Oil Mill Effluent (POME). The incorporation of 2% silver-coated HA demonstrated a rejection efficiency of 89.74% and possessed exceptional separation characteristics. The incorporation of a filler comprising hyaluronic acid and boron nitride into a polyethersulfone/polyvinylpyrrolidone membrane resulted in a two-fold increase in flow, while achieving a 90% rejection rate of bovine serum albumin solution [21]. Mu et al. [19] examined the effect of hydroxyapatite nanotubes on the hydrophilicity of PES/PVP membranes. The HA nanotubes were coated with PDA, subsequently followed by taurine grafting. This intricate change enabled the engineered membranes to achieve enhanced permeability, a 97% rejection of BSA, and improved antifouling characteristics.

This research examines the impact of adding hydroxyapatite (HA) to a Polysulfone (Psf) dope solution to produce ultrafiltration membranes. The purchased HA was utilized without alteration to prevent additional production expenses. The objective was to improve the membrane selectivity characteristics while preserving elevated permeability at the ultrafiltration level. The membrane was made using the non-solvent-induced phase separation (NIPS) technique. The influence of hydroxyapatite on the membranes’ surface morphology, membrane pore characteristics, permeate flux, and hydrophilicity membrane was analyzed. Scanning Electron Microscopy (SEM) was used to visually study the membrane surface morphology. To evaluate the membranes’ efficacy in protein separation, the fabricated Psf membranes were subjected to aqueous protein solutions of different molecular weights, and the protein retention percentages of the membranes were measured.

## 2. Materials and Methods

### 2.1. Materials

The polymer used in the study was Polysulfone (Psf), with a molecular weight of 78,000 to 84,000 g/mol (P.T. Solvay Chemical, Cilegon, Indonesia). The solvent, NMethyl-2-Pyrrolidone (NMP), was purchased from Merck KGaA, Darmstadt, Germany. Hydroxyapatite powder (HA) with a molecular weight of 502.31 g/mol and a purity of at least 90% was purchased from Sigma Aldrich, St. Louis, MO, USA. Additionally, Bovine serum albumin (BSA) and lysozyme were acquired from HiMedia Ltd., Mumbai, India. Pure water was utilized throughout the whole fabrication process. All chemicals, unless stated otherwise, were of analytical grade with over 98% purity and were used as received.

### 2.2. Membrane Fabrication

The ultrafiltration membranes were fabricated using the non-solvent-induced phase separation (NIPS) technique, a recognized method used to produce asymmetric membranes [22]. Initially, a solution comprising Psf as the base polymer and NMP as the solvent was continuously stirred at 70 °C until a homogeneous solution was obtained. The dope solution was maintained at a consistent 20 wt.% concentration of Polysulfone. Thereafter, modest amounts of HA particles (Sigma Aldrich) were added into the solution as a membrane additive. The Psf solution was subsequently combined with 0.1 wt.% to 0.5 wt.% of HA particles, heated, and agitated at 70 °C for 3 h to produce homogeneous dope solutions. Following this procedure, the dope solutions were then cooled to room temperature naturally for the membrane fabrication procedure. Subsequent to cooling, the dope solution was deposited onto a glass plate and evenly distributed with a membrane applicator (M43 6BU, Elcometer Ltd., Manchester, UK) to attain a consistent wet thickness of 200 μm. The glass plate containing the solution was carefully placed in a pure water container to commence the gelatinization process. Subsequently, the membrane was submerged in distilled water for 24 h prior to testing. The membranes were designated according to their HA content: HA-0.0 (native polysulfone), HA-0.1, HA-0.2, HA-0.3, HA-0.4, and HA-0.5.

### 2.3. Membrane Characterization

**Figure 1:**
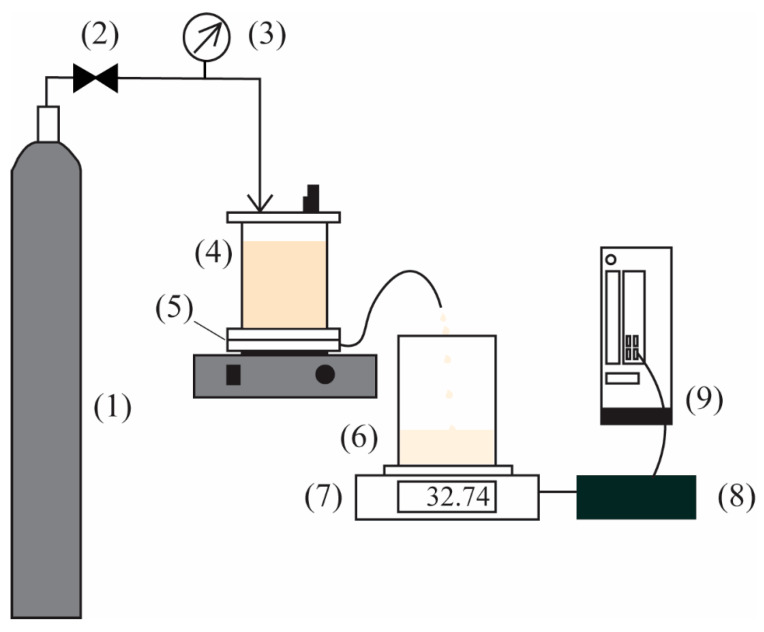
The schematic diagram of the dead-end cell filtration. (1) Nitrogen gas cylinder; (2) Pressure regulator; (3) Pressure gauge; (4) Dead-end cell; (5) Membrane; (6) Permeated water beaker; (7) Electronic weight balance; (8) Weighing environment logger; (9) Computer.

This work assessed membrane effectiveness using pure water as the testing fluid. The test for each membrane lasted 30 min to reach steady flux in a stirred dead-end cell (HP4750, Sterlitech Corp., Kent, WA, USA). The effective membrane area used for filtration was 13.4 cm^2^, as depicted in Figure 1. Nitrogen gas with a fixed pressure of 2 bars was introduced to the pure water chamber in order to ensure that consistent pressure was applied throughout the test.

During the test, the weight of the permeate water that traversed the examined membrane was logged using a Weighing Environment Logger (AD-1687, A&D, RoHS, Tokey, Japan). The volumetric flux ($J_{v}$) and permeability flux ($L_{p}$) were computed utilizing the following formulas [23]:

(1) $$J_{v}=\frac{Q}{A\times \Delta t}$$

(2) $$L_{p}=\frac{J_{v}}{\Delta P}$$

where Q represents the volume of the penetrated water collected throughout the collecting period, Δt denotes the collection period, A signifies the membrane, and ΔP indicates the pressure differential.

Subsequent to the permeate flux experiment, a membrane selectivity assessment was executed utilizing a 1 g/L protein solution under the same conditions. To assess the protein separation, Bovine Serum Albumin (BSA, Mw = 66.5 kDa) and lysozyme (Mw = 14.3 kDa), which serves as a representative of α-Lactalbumin, were utilized. The proteins were formulated in a phosphate-buffered solution at a pH of 7.2. The protein separation experiment was conducted in a dead-end cell filtration system, maintaining a constant pressure of 2 bar. The protein concentration in the permeated solutions was measured using an N4S UV-Visible Spectrophotometer (Ningbo Hinotek Instrument Co., Ltd., Ningbo, China), in which the wavelength was set to 280 nm. Solute rejection (SR) was evaluated using the following formula [24]:

(3) $$\%SR=\left[1-\frac{C_{p}}{C_{f}}\right]\times 100$$

where $C_{p}$ and $C_{f}$ denote the concentrations of protein in the permeating and feed solutions, respectively. The data presented in this study reflect the mean values from three samples per membrane.

Five iterations of BSA tests were conducted to evaluate the antifouling efficacy of the membranes. Each cycle comprises a 30 min water flux followed by a 10 min BSA flux. The flux decline ratio (FDR) and flux recovery ratio were determined by [25]:

(4) $$FDR=\frac{J_{v}-J_{vt}}{J_{v}}\times 100\%$$

(5) $$FRR=\frac{J_{v\prime }}{J_{v}}\times 100\%$$

where $J_{vt}$ is the permeate flux at the end of the fifth cycle, and $J_{v\prime }$ is the permeate flux of the second cycle. A lower FDR value signifies the superior adhesion resistance of foulants or the enhanced antifouling performance of the membrane. A higher FRR value denotes improved efficiency in membrane cleaning.

The hydrophilicity of the membrane was assessed by determining the angle between a water droplet and the membrane surface. Then, 5 µL of distilled water was dropped carefully onto the top of the membrane surface using a micropipette. A digital microscope (Dinolite Edge 3.0 AM73915MZTL) subsequently recorded an image of the contact angle of the water droplet. The angle was subsequently gauged using AutoCAD software (v2022). To guarantee precision, the contact angles were assessed four times at random spots of the membrane surface, and the average value was determined. The morphologies of the membrane surfaces and their cross-sectional structures were examined utilizing scanning electron microscopy (SEM Phenom ProX, ThermoFischer, Waltham, MA, USA). The cross-sectional micrographs were obtained by cracking wet membrane samples subsequent to their rapid freezing in liquid nitrogen.

### 2.4. Pore Characterization

To evaluate the porosity of the membrane surface, the dry membranes were initially trimmed to a predetermined size, then soaked in glycerol for 24 h. The glycerol was further extracted to weigh the wet membrane ($W_{w}$). The moist membrane was thereafter dried in a desiccator and allowed to desiccate for one day to acquire the weight of the dry membrane ($W_{d}$). The porosity of the membrane surface was examined to evaluate the effect of hydroxyapatite incorporation on the membrane pore size. This investigation was performed utilizing a gravimetric approach, which involves the following formula [26]:

(6) $$\varepsilon \left(\%\right)=\frac{W_{w}-W_{d}}{\rho H_{2}O\times A\times L}\times 100$$

where A represents the effective filtration area of the membrane, L denotes the thickness of the membrane, and $\rho H_{2}O$ signifies the density of the water (0.998 g/cm^3^). The average pore size ($r_{m}$) of the prepared membranes can be determined using the Guerout–Elford–Ferry Equation, including the porosity data and permeate flux [27]:

(7) $$r_{m}=\sqrt{\frac{\left(2.9-1.75\varepsilon \right)8\mu_{H_{2}O}\times L\times Q_{H_{2}O}}{\varepsilon \times A\times \Delta P}}$$

where ε denotes the porosity of the membrane, $\mu_{H_{2}O}$ represents the dynamic viscosity of water at an ambient temperature, L indicates the membrane thickness, $Q_{H_{2}O}$ is the volume of water traversing through the membrane per unit time, A refers to the active filtration area of the membrane, and ΔP is the transmembrane pressure.

## 3. Results and Discussion

### 3.1. Membrane Morphology

Five membrane samples were fabricated with differing amounts of hydroxyapatite. Among this collection of fabricated membranes, one was designated as HA-0.0, which functioned as a reference without the inclusion of HA. The remaining membranes were designated as HA-0.1, HA-0.2, HA-0.3, HA-0.4, and HA-0.5, reflecting the HA weight content in the Psf dope solution. The whole samples were fabricated with a wet thickness of 200 μm. The surface morphologies of the membranes were analyzed using a SEM Phenom ProX microscope.

**Figure 2:**
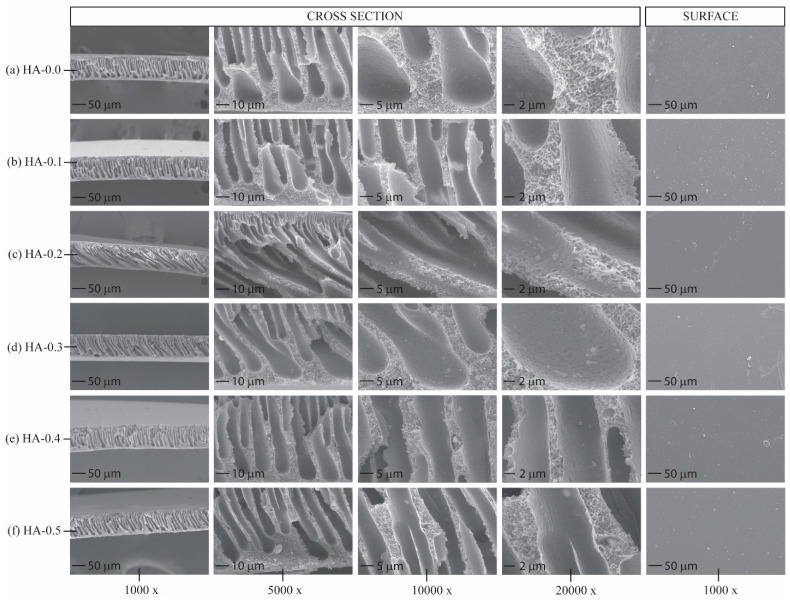
Morphologies of membrane samples. Cross-sectional images at both low and high magnification are displayed in the first to fourth rows, while the fifth row features top surface micrographs: (**a**) HA-0.0; (**b**) HA-0.1; (**c**) HA-0.2; (**d**) HA-0.3; (**e**) HA-0.4; (**f**) HA-0.5.

Cross-sectional scanning electron microscopy (SEM) examination is a prevalent method for visually assessing membrane morphology. Figure 2 presents the cross-sectional micrographs of the native Psf membrane and the Psf/HA blend membranes, with magnifications varying from 1000× to 20,000× throughout the first to fourth rows. The fifth row displays the micrographs of the membrane surface. At 1000× magnification, all membranes have a thin surface layer atop a vertically extending, finger-like intermediate structure. The lower section of the membrane exhibits a sparse, spongiform architecture characterized by discernible wall pores. In the membrane gelatinization phase of the NIPS technique, the interaction between NMP as the solvent and pure water as the non-solvent induces solidification due to the thermodynamic instability of the cast solution [28]. The cast solution quickly interfaces with water, resulting in immediate vitrification and permitting water ingress, which generates macrovoids inside the membrane structure. Hackett et al. [29] explored how the composition of the coagulation bath impacts the efficacy of the Psf membrane. They found that utilizing solely pure water during membrane submersion retains a significant amount of solvent in the central region due to NMP–water incompatibility. Kang et al. [30] discovered that blending hydrophilic agents into the membrane dope solution, such as silica or HA, improves the interchange rate of solvent and non-solvent throughout the solidification phase, thus enlarging the macrovoids at deeper depths, which mimic the form of pre-inflated balloons. The lower section of the membrane exhibits a distinct coagulation rate when water permeates from below; this is attributable to the glass support, and leads to the formation of spongy structures with bigger pores akin to those on the upper surface.

The SEM cross-sectional micrographs demonstrate the existence of hydroxyapatite (HA) on the surface of finger-like structures and within wall pores, exhibiting a random distribution from the top region to the bottom region of the membrane. HA is also present on the surface and affixed irregularly, resulting in surface protuberances. The SEM cross-sectional views reveal no detectable alterations in the dimensions of the membrane wall pores or macrovoids attributable to HA. Nonetheless, the blend membranes possess HA globules that are randomly affixed to the macrovoid surfaces. The variation in globule diameters indicates the aggregation of HA particles during gelatinization. The heightened viscosity of the Psf blend membranes, resulting from the enhanced HA concentration, influences the morphology of the Psf/HA blend membranes. Certain particles infiltrate the wall pores, becoming trapped within the membrane. A greater quantity of HA particles is clearly detected at the membrane’s base, especially in HA-0.5, which is attributable to the gravitational settling and agglomeration of HA particles during the casting procedure [31,32]. The zeolite-nanoparticle-impregnated Psf membrane exhibited particle agglomeration at an elevated loading level [33]. Yurekli further indicated that the membrane might achieve a saturation point at which the maximal loading of zeolite nanoparticles was reached.

### 3.2. Water Contact Angle Analysis

A primary disadvantage of Psf membranes is the accumulation of build-up on their surface, leading to pore obstruction [31]; thus, hydrophilicity is crucial for membrane efficacy. The measurement of the water contact angle is frequently applied to evaluate the hydrophilicity and wettability of membrane surfaces. This method assesses the angle formed between the water droplet and a planar membrane surface, signifying hydrophilicity when the angle is between 0° to 90°, or hydrophobicity when the angle is larger than 90°. A decrease in the contact angle with water indicates an enhancement in the membrane’s hydrophilic characteristics [34]. If the contact angle is below 90°, the liquid adheres to the surface, and at 0 degrees, the liquid uniformly disperses throughout the surface.

**Figure 3:**
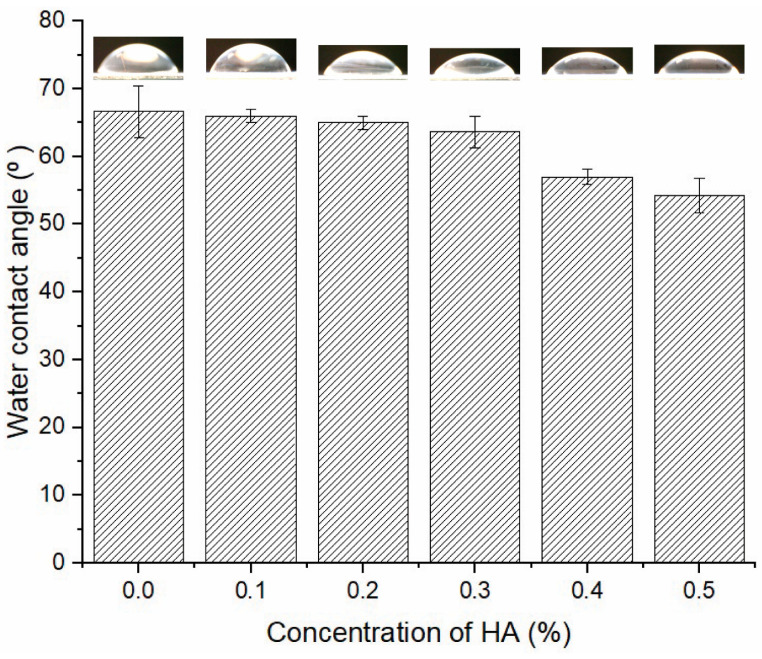
Water contact angle of Psf membranes with varied hydroxyapatite concentrations.

Figure 3 illustrates the measurement of water contact angles for different concentrations of HA. The native Psf membrane examined in this study exhibits a contact angle of 66°, aligning with previous results indicating that native Psf membranes possess water contact angles ranging from 60° to 70° [29,33,34,35]. Figure 3 indicates that hydrophilicity enhances with an elevated HA content. The Psf/HA-0.5 membrane had the highest hydrophilicity, with a contact angle of 54°, whereas the contact angles of Psf/HA-0.1 to 0.3 were comparable to that of the native membrane. This tendency indicates that elevated HA concentrations increase hydrophilicity owing to the hydroxyl groups on the particle surfaces that facilitate hydrogen bonding to the sulfonic groups of Psf [33,36]. During the phase separation process, the hydrophilic agent will draw in molecules of water, creating more spaces for water infiltration [37]. SEM micrographs of the composite membrane surface revealed a random distribution of HA particles. Due to its hydrophilic nature, HA tends to attract and engage with water rather than repel it. Astala and Stott [38] explained that HA surfaces are distinguished by undercoordinated oxygen atoms in surface phosphate groups. A key process for water adsorption entails the interaction between O atoms and H_2_O molecules through hydrogen, with Ca-rich surfaces exhibiting the highest reactivity. Most studies propose that the enhancement in membrane hydrophilicity is directly linked to the existence of hydrophilic nanofillers located near or spread on the surface of the membrane [39].

### 3.3. Permeate Flux Test Experiments

**Figure 4:**
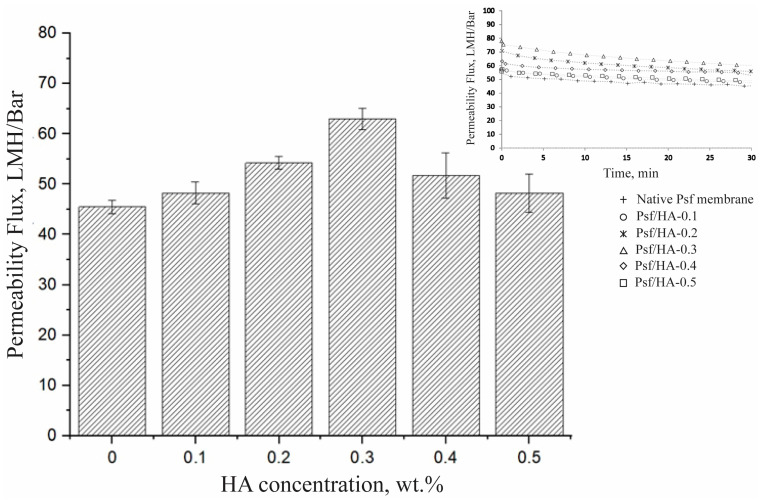
Permeability flux of Psf membranes with varied HA concentrations.

Figure 4 presents the permeate flux of Psf/HA blend membranes conducted in a dead-end filtration cell at a pressure of 2 bar. The permeate flux was observed for 30 min, repeated four times, and the average was determined. The permeate flux value utilized for computation was documented following 30 min of filtering. The native Psf membrane demonstrated a permeate flux of 45.5 LMH/bar. The permeate flux augmented with HA concentration, attaining a peak flux of 63 LMH/bar at 0.3 wt.%, then declining to a value comparable to that of the native Psf membrane. The trend in the permeate flux demonstrated that flux rises with the HA content up to 0.3 wt.% and thereafter declines, nearing the value of the native membrane. This reduction is probably attributable to the existence of HA. At reduced concentrations, HA particles generate open voids by infiltrating wall pores, hence augmenting the flux. At concentrations beyond 0.3 wt.%, the surplus HA particles obstruct the pores, hence diminishing water movement. Eslami et al. [31] attribute the diminished flux values to a surge in viscosity, which subsequently reduces the solvent/non-solvent exchange rate, resulting in lower flux values.

### 3.4. Protein Separation Performance Analysis

Ultrafiltration is the predominant industrial technique used for isolating whey proteins. The extent of protein adherence on the surface of the membrane profoundly influences the assessment of anti-fouling capacity. The reduced adsorption of protein results in enhanced membrane resistance to fouling. Proteins often exhibit stronger adsorption on hydrophobic surfaces and are less easily desorbed compared to hydrophilic surfaces. Cayot and Lorient [40] assert that α-Lactalbumin is the smallest predominant whey protein, possessing a molecular weight of 14 kDa and comprising around 20–25% of whey protein. The predominant whey protein is Immunoglobulins, possessing a molecular weight of 150–1000 kDa and comprising approximately 10% of whey protein.

**Figure 5:**
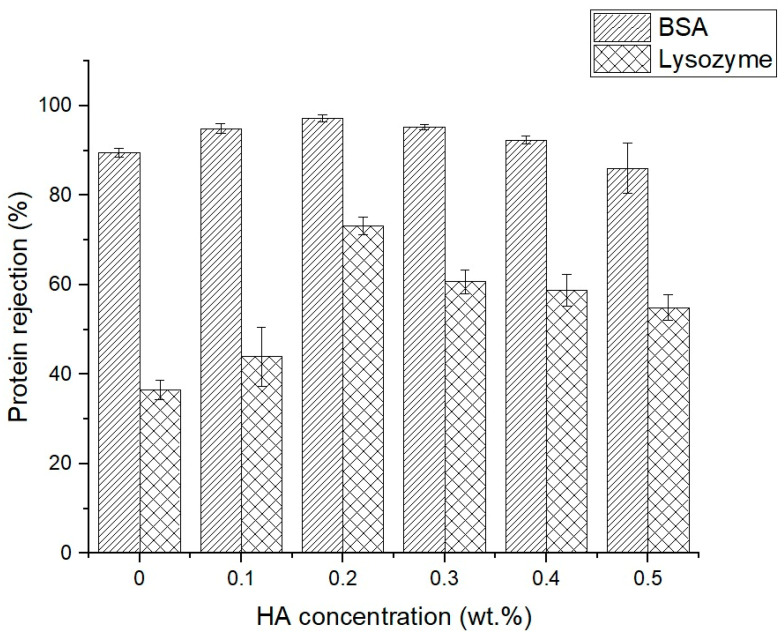
Protein rejection of Psf membranes with varied HA concentrations.

All membranes were initially compressed at a 2 bar pressure with pure water for 30 min prior to testing. Figure 5 illustrates the protein separation of BSA, a large protein with a molecular weight of 66.5 kDa, and lysozyme, a tiny protein (14.3 kDa), which signifies α-Lactalbumin. Upon BSA rejection, the rejection rate of all membranes exceeds 85%. The native Psf membrane exhibits a BSA rejection rate of 89.6%, which increases to 97.2% with the incorporation of HA at a concentration of 0.2 wt% in Psf/HA blend membranes. The rejection diminishes beyond this juncture. Lysozyme rejection demonstrates considerable enhancement with a reduced protein size. The native Psf membrane exhibits a rejection rate of 36%, while the blend membranes demonstrate enhanced rejection capabilities. The peak rejection is 0.2 wt.% at 73% (double-fold value of the native Psf membrane), and the rejection diminishes to 54% at HA 0.5 wt.%.

**Figure 6:**
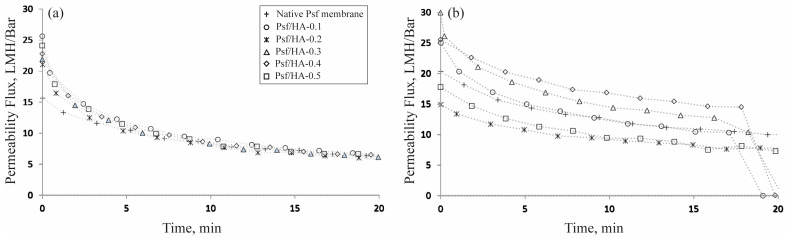
Protein flux of blend membranes: (**a**) BSA flux; (**b**) Lysozyme flux.

Protein flow was observed for 20 min for both BSA and lysozyme, each at a concentration of 1000 mg/L (Figure 6). The BSA flux exhibited comparable patterns and values for both the native and blend membranes. The fluxes of the Psf blend membranes were initially marginally different to those of the native membrane but progressively diminished to align with the flux of the native membrane. This reduction is presumably because of BSA molecules occupying the membrane’s interstices, progressively obstructing them over time. At the conclusion of the experiment, all membranes had comparable flux levels.

The flux of lysozyme across the native membrane exceeded that of BSA, presumably because lysozyme has a smaller molecular size than BSA. All membranes exhibited a more gradual reduction in lysozyme flux relative to BSA flux, which is ascribed to the smaller molecular size of lysozyme. The native membrane exhibited a lysozyme rejection rate of merely 36.5%, which increased to 73.2% upon elevating the HA concentration to 0.2 wt.%. The enhancement in lysozyme rejection is attributed to protein adsorption by HA across the membrane. Nevertheless, as the HA concentration was elevated, lysozyme rejection diminished by as much as 26% at 0.5 wt.% HA. This decrease is probably attributed to inadequate space for the liquid to flow, as the majority of open holes were occupied by HA particles. Both 0.2 and 0.5 wt.% HA exhibited reduced fluxes, maybe attributable to distinct factors. At 0.2 wt.%, the reduced flux was attributable to the adsorption of lysozyme by HA, leading to optimal rejection. At 0.5 wt.%, the diminished flux was attributed to the open spaces being obstructed by HA powder, impeding liquid flow through the membrane, thereby leading to reduced flux and simultaneous rejection. Salimi et al. [41] further indicated that augmenting the HA content in the membranes improved BSA rejection. The incorporation of a higher HA concentration led to a thicker membrane skin layer, smaller pore size, and lower permeation flux.

### 3.5. Membrane Pore Analysis

Table 1 outlines the surface porosity and mean pore size of the native Psf and blend membranes. Notably, the porosity of the blend membranes, with the exception of Psf/HA-0.5, is inferior to that of the original Psf membrane. As HA comprises solid particles that remain undissolved, it is anticipated that the membrane will grow increasingly compact with higher HA concentrations. The porosity values ranging from 0.1 wt.% to 0.4 wt.% are comparable; however, the porosity at 0.5 wt.% exhibits a considerable rise, exceeding that of the native Psf membrane. The equation for determining porosity incorporates the wet and dry membrane weights, the effective membrane area, and the membrane thickness. A significant rise in membrane thickness was noted during measurement, possibly attributable to the substantial existence of HA on the surface of the membrane. This presence influences the thickness measurement, resulting in a calculation that suggests the membrane is more porous. Nevertheless, according to the water flux and protein separation measurements, the porosity of Psf/HA-0.5 should indeed be smaller. The size of macropores within the finger-like structures also influenced the membrane porosity value. The visible macropores were quantified using the SEM images for each sample. The macropore sizes are somewhat comparable, with Psf/HA-0.2 exhibiting the smallest macropore size among all samples examined. The flux recovery ratio (FRR) was calculated using Equation (5). A higher FRR value indicates enhanced efficiency in membrane cleaning [25], which in this case was when 0.1 wt.% HA was applied. All blend membranes, with the exception of Psf/HA-0.5, exhibit a better membrane cleaning efficiency relative to the native Psf membrane.

**Figure 7:**
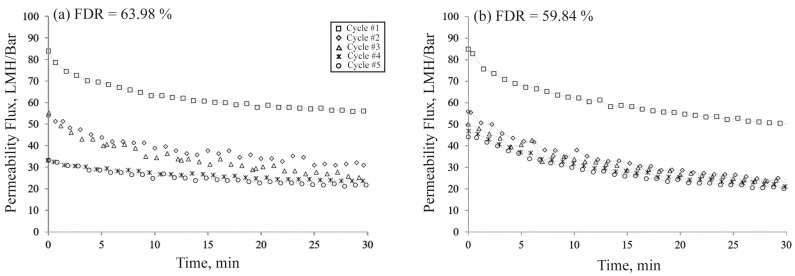
Flux decline ratio (FDR); (**a**) Native Psf membrane; (**b**) Psf/HA-0.2.

Figure 5 illustrates that the Psf/HA-0.2 membrane demonstrated the highest protein rejection. To evaluate the effect of HA on the membrane lifespan, we performed five cycles of protein filtration, each consisting of a 30 min pure water flux test followed by a 10 min BSA filtration. Upon completion of the test, the flux decline ratio (FDR) could be determined in accordance with Equation (4). A lower FDR value generally indicates that the membrane exhibits more resistance to foulants, signifying enhanced antifouling properties [25]. Figure 7 illustrates that, while the flux trends for both the native Psf membrane and the blend membrane were comparable during the first and second cycles, the third cycle and subsequent cycles exhibited distinct values and trends. The native membrane exhibited a flatter flow pattern, indicating the significant onset of pore blockage. In the blend membranes, the beginning fluxes consistently exceeded the final fluxes over all five cycles. Despite the reduction in the flux value with each cycle, the downward trend in flux suggested that the amount of foulants adhering to the membrane surface was less than that of the native membrane. The incorporation of HA into the Psf membrane enhanced its hydrophilicity, which is advantageous for the whey separation industry.

Table 2 compares prior studies on membrane performance that utilized HA as the primary modulator. Each column presents the value of the natural membrane in comparison to its modified counterpart. A variety of polymer-based solutions were utilized, including polyethersulfone (PES), polysulfone (PSf), and polyphenylsulfone (PPSU). While most studies employed PVP as a pore former, this experiment did not utilize PVP, hence influencing porosity and flux. Besides employing PVP as a pore former, HA was also modified by coating and grafting techniques. Each approach enhances the flux, pore size, rejection, and hydrophilicity, facilitating whey protein separation.

The optimal membrane for this study exhibits a performance equivalent to earlier research on protein rejection and hydrophilicity, despite the hydroxyapatite not undergoing any modifications before usage. Future research should utilize pore-forming agents, such as PVP, to enhance flow. The straightforward membrane fabrication procedure presented in this study should be the focal point for scaling up membrane production in the dairy industry.

## 4. Conclusions

The performance of polysulfone (Psf) ultrafiltration membranes was investigated after modification with hydroxyapatite (HA). These flat-sheet membranes were produced using the wet-phase inversion method. Scanning Electron Microscopy (SEM) analysis revealed that the HA particles were randomly distributed throughout the membrane. The surface hydrophilicity increased with HA concentration due to hydrogen bonding with HA’s hydrophilic properties. The highest water flux was observed at 0.3 wt.% HA, but it decreased at higher concentrations, likely due to pore blockage by HA particles. Both native and modified membranes showed high Bovine Serum Albumin (BSA) rejection, with the highest rejection at 0.2 wt.% HA. A significant improvement was noted in lysozyme separation, with the maximum rejection increasing from 36.5% in the native membrane to 73.2% in the modified membrane. This enhancement is attributed to protein adsorption by HA. Contact angle measurements validate the enhanced hydrophilicity of the Psf membrane with increasing HA concentration. This cost-effective and facile approach to enhancing permeability while maintaining the separation efficiency of the membrane has the potential to scale up industrial application.

## Figures and Tables

**Table 1:** Surface porosity, pore size, and FRR of Psf/HA blend membranes.

Membrane Code	Porosity, ε (%)	Mean Pore Size, *r_m_* (Å)	Macropores Size, µm	FRR, (%)
Psf/HA-0.0	68.91	42.41	0.44 ± 0.093	48.72 ± 7.558
Psf/HA-0.1	65.72	40.04	0.48 ± 0.090	79.6 ± 4.845
Psf/HA-0.2	65.28	38.97	0.42 ± 0.074	73.2 ± 4.663
Psf/HA-0.3	66.28	39.91	0.49 ± 0.066	69.05 ± 4.621
Psf/HA-0.4	66.92	41.12	0.49 ± 0.055	68.70 ± 6.603
Psf/HA-0.5	71.01	43.82	0.49 ± 0.020	30.23 ± 6.999

**Table 2:** Overview of research on HA blending in polymeric membranes comparing the native membrane and modified membranes.

Polymer	Modifier	Permeability Flux, LMH/Bar	Pore Size, nm	Rejection (%)	ε (%)	WCA (°)	Ref.
PES/PVP	HA nanotubes–PDA coating–taurine grafting	169/439	34.1/43.2	~97 (BSA)	55/73	70/53	[19]
PPSU	Silver-coated HA	39/57	-	89.74 (oil)	-	-	[20]
PES/PVP	HA/boron nitride	286/620	54/66.7	90 (BSA)	-	-	[21]
PES/PVP	HA	67/148.7	81/84	97.3 (BSA)	-	-	[41]
Psf/PVP	HA-modified PDA and PEI	152.8/481.6	5.36/7.41	94.5 (BSA)	52/74	81/60	[42]
Psf	HA	45.5/63	4.24/3.89	97.2 (BSA) 73.2 (Lysozyme)	68/65	66/65	This work

## Data Availability

The original contributions presented in the study are included in the article, further inquiries can be directed to the corresponding author.
